# Technical architecture for integrating vision-language models with DICOM viewers

**DOI:** 10.1186/s41747-026-00769-0

**Published:** 2026-07-01

**Authors:** Marco Albera, Anna Colarieti, Irene Albera, Alessandro Carriero

**Affiliations:** 1 SCDU Radiodiagnostica, Ospedale Maggiore Della Carità, Novara, Italy; 2 Università degli Studi del Piemonte Orientale “Amedeo Avogadro”, Novara, Italy

**Keywords:** Artificial intelligence, Computer graphics, DICOM, Radiology workflow, Vision-language models

## Abstract

**Abstract:**

Integrating vision-language models (VLMs) into clinical radiology workflows requires exporting two-dimensional images that preserve diagnostic viewing context, including imaging plane, slice position, window/level, slab parameters, and overlays. Existing approaches typically rely on *ad hoc* screenshots and external uploads, which discard this context, reduce reproducibility, and introduce workflow friction through task switching. We describe a radiology viewer architecture that maintains model interaction entirely within the diagnostic environment by serializing explicit viewer state into replayable descriptors. Requested views are re-rendered offscreen using shared in-memory voxel data, enabling repeatable, context-faithful evidence generation without disrupting interactive interpretation. The user interface employs a split workspace, combining a diagnostic grid for image review with an integrated chat sidebar for evidence staging and model interaction. Capture actions generate batches of serialized viewer states that are executed by an offscreen renderer; the resulting frames are staged for user curation prior to model submission. Under controlled conditions at a 1,000 × 1,000 pixel output resolution, benchmarks demonstrated perfect reproducibility: repeated captures from identical serialized states produced pixel-identical images, with zero hash mismatches across 50 frames. Capture latency ranged from a median of 4.49 to 16.69 ms per frame, depending on view type, and median output size was 27.9 kB per frame using a high-efficiency image container. These results indicate that the proposed architecture supports reproducible, low-latency, and storage-efficient multislice and multiseries evidence assembly at interactive speeds, enabling seamless integration of VLMs into diagnostic radiology workflows.

**Relevance statement:**

Offscreen re-rendering driven by serialized viewing state enables context-preserving and reproducible export of two-dimensional evidence from volumetric radiology viewing, supporting integrated vision-language model workflows with millisecond-scale capture latency from hot cache.

**Key Points:**

- A split-layout DICOM viewer with an integrated chat interface enables radiologists to capture, stage, and submit visual evidence without leaving the diagnostic environment.
- Offscreen re-rendering from serialized viewing state produces pixel-identical exports across repeated captures, supporting traceable, auditable, and reproducible evidence generation.
- Batch capture efficiently assembles multislice, multiplane, and multiseries evidence with minimal manual effort, providing models with richer and more representative study context.
- Capture latency was millisecond-scale, and high-efficiency image container (HEIC) encoding reduced file size by approximately 2.8-fold compared with the joint photographic experts group (JPEG) format, supporting responsive multi-frame workflows.

**Graphical Abstract:**

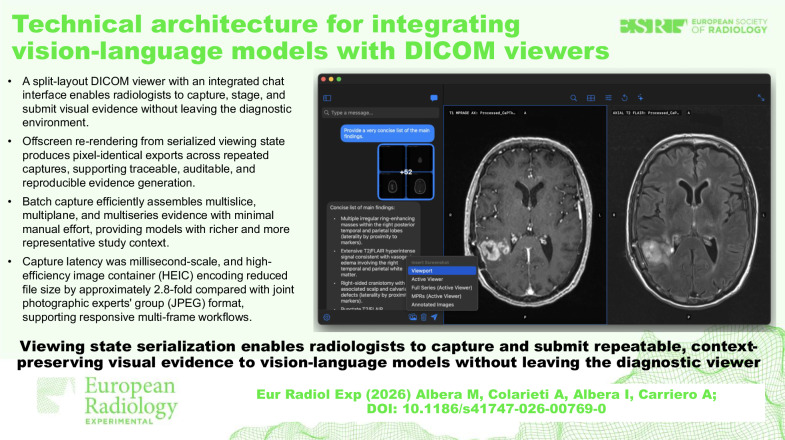

## Background

Vision-language models (VLMs) represent a major advancement in artificial intelligence, evolving from text-only large language models (LLMs) to “multimodal” systems capable of analyzing text and images simultaneously [1, 2]. For medical imaging, this marks a significant paradigm shift. Historically, traditional “narrow” computer vision tools in radiology were designed to perform single, rigid tasks, such as placing bounding boxes around lesions or measuring volumes *via* segmentations. While useful, these tools cannot answer spontaneous clinical questions or adapt to complex contexts.

In contrast, VLMs act more like generalist clinical assistants. Because they accept both visual input (images) and natural language prompts (text), they can answer open-ended questions about what they see. For example, a radiologist could highlight a complex hepatic mass and ask, “Does the enhancement pattern of this lesion align better with hepatocellular carcinoma or a metastasis, given the patient’s history of melanoma?” The VLM can then synthesize the visual evidence with the clinical background to provide differential diagnoses, suggest next steps, or help draft the report impression.

However, for VLMs to effectively support radiology workflows, they must be grounded in the exact same visual evidence the radiologist is reviewing [3, 4]. In practice, this creates a major technical bottleneck: most VLM endpoints are designed to accept standard, two-dimensional images (like JPEGs), while diagnostic interpretation in radiology is inherently volumetric, interactive, and stored in the Digital Imaging and Communications in Medicine (DICOM) format. A clinically meaningful “view” is defined not only by pixels, but by plane and slice selection, window and level, slab type and thickness, camera geometry, and overlays or annotations that may encode interpretive intent.

The two most common ways radiologists currently interact with VLMs occur outside the diagnostic viewer, creating workflow friction by forcing users to switch applications and perform ancillary steps [5]. One approach is text-only prompting, where radiologists describe imaging findings in free text without sharing the underlying images; this requires translating complex visual information into language, which can omit subtle but clinically important details and leaves the model disconnected from the actual visual evidence [6, 7]. The second approach is manual image export and upload, typically using screenshots or selected key images captured from the viewer; while this preserves some visual context, it reduces a volumetric, interactive examination to a limited set of static images that may not retain critical information such as slice location, imaging plane, window and level settings, slab configuration, camera orientation, or visible overlays and annotations. As a result, the evidence provided to the model may be incomplete, difficult to reproduce, and challenging to audit or evaluate retrospectively.

This paper describes a viewer-centric integration approach where volumetric context capture is treated as a first-class workflow and model interaction remains within the viewer rather than pushing users into separate export-and-upload steps. Rather than relying on *ad hoc* viewport screenshots or acquiring the current display buffer as-is, this novel system serializes explicit viewing state, re-renders the requested contexts offscreen, and stages the generated frames directly in an embedded chat sidebar so that evidence selection and question formulation remain tightly coupled without disrupting the diagnostic grid. Because exported evidence is derived from an explicit, replayable state, capture can be logged, repeated, and evaluated in deployment.

## Methods

### System overview and user workflow

**Fig. 1:**
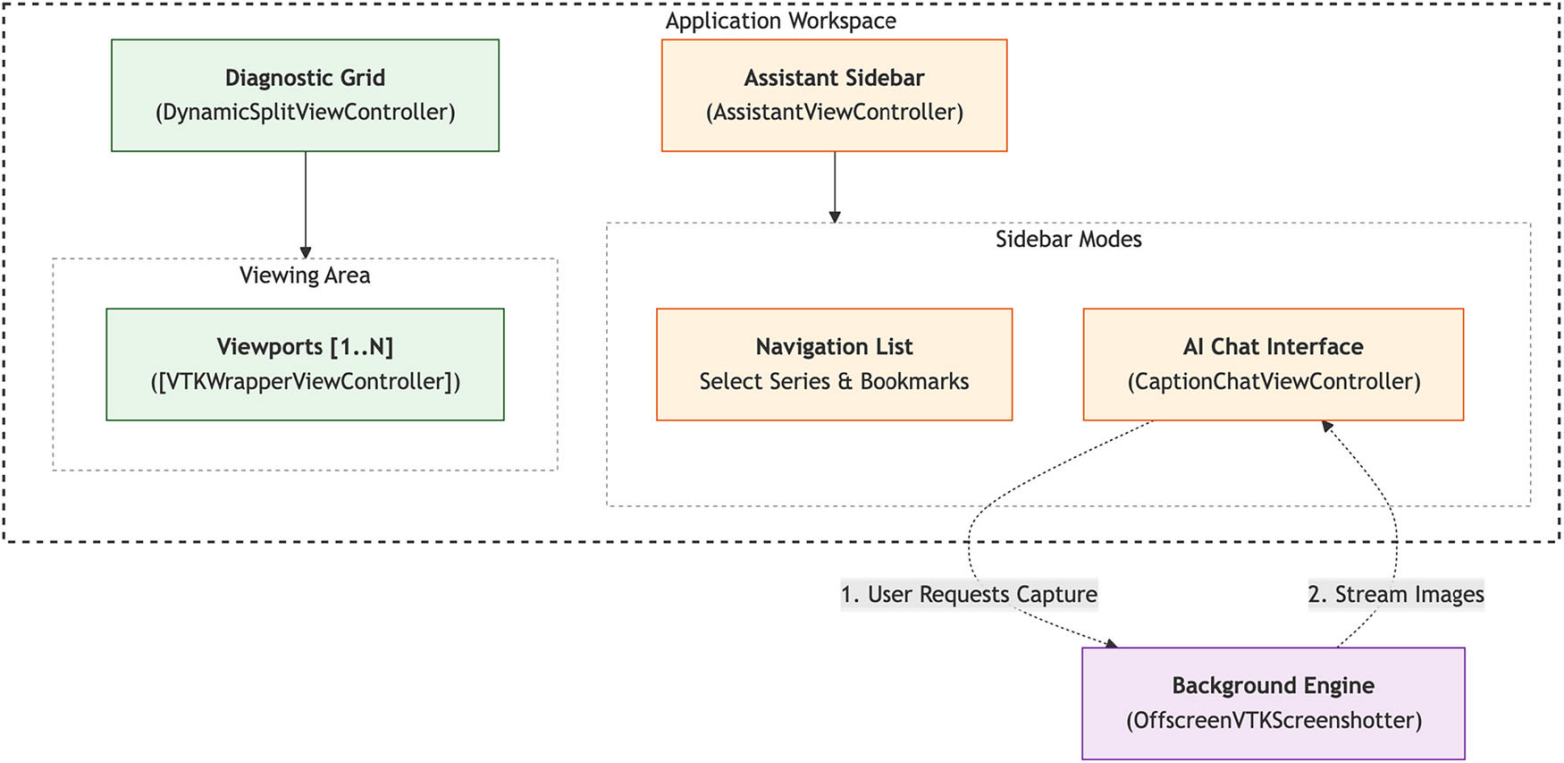
High-level architecture. The viewer is organized as a split workspace with a Diagnostic Grid for interactive interpretation and an Assistant Sidebar for navigation, evidence staging, and chat. Capture actions do not screenshot displayed pixels; instead, they enqueue explicit, serialized viewer-state descriptors (series, mode, plane intent, slice selection, window/level, slab settings, camera when needed, and overlay policy). A cloned offscreen viewer executes the queued states using shared in-memory voxel data, re-renders each requested context from serialized state, and returns frames incrementally to the sidebar for curation prior to submission to a model endpoint. Provider-specific model wiring is isolated to a thin adapter layer that packages selected images and parses responses

The application presents a split workspace with a diagnostic grid for interactive interpretation and an assistant sidebar for navigation, evidence staging, and chat (Fig. 1), using UIKit to support a single codebase across iOS and macOS. The current implementation targets the Apple ecosystem, but the architectural approach is portable because viewing-state serialization and offscreen re-rendering are framework-agnostic. Evidence capture can be initiated from an active viewport or from a series list. Rather than screenshotting displayed pixels, each request is converted into one or more explicit viewer-state descriptors. A separate offscreen viewer executes those states using shared in-memory imaging data and returns exported frames to the sidebar for user selection before model submission. Terminology is summarized in Supplementary Chapter S3.

### Viewing modes and overlay layering

Viewing modes include two-dimensional slice navigation and multiplanar reconstruction (MPR) in axial, coronal, and sagittal orientations, with optional slab rendering. Overlays are drawn in a separate layer and may be excluded or composited at export according to policy; layering details are provided in Supplementary Chapter S3.

### Viewer state serialization

An exportable view is represented by a serialized viewer state that encodes the parameters needed to reproduce a frame. Interactive viewports can serialize their current state, while batch actions generate state sequences such as sampled slices or orthogonal plane sets. Field definitions and sampling policies are described in Supplementary Chapter S1.

### Offscreen re-rendering and shared imaging data

Export is performed by a separate offscreen viewer instance that applies each queued state and re-renders the requested context. This isolates capture from interactive viewing and associates each exported frame with a replayable state. The offscreen instance reuses shared in-memory imaging data while keeping visualization settings independent. Loading and request management details are provided in Supplementary Chapter S3.

### Overlay compositing and protected health information policy

Overlays may carry clinical meaning and, when enabled, can be burned into exported pixels. Because overlays may also contain identifying text, export is policy-controlled and can be disabled. Exported images exclude DICOM metadata, and deployments must ensure identifiers do not appear in exported pixels or filenames. The current implementation does not perform automated burned-in-text detection; prevention relies on deployment-level overlay configuration and institutional policy. Additional policy details are provided in Supplementary Chapter S3.

### Graphics capture, encoding, and request/response semantics

Offscreen capture renders to a graphics processing unit-backed target and exports a pixel buffer for optional overlay compositing and image compression. Frames are encoded in model-compatible formats such as joint photographic experts group (JPEG) or high-efficiency image container (HEIC) in the viewer’s display orientation. Images are staged locally and submitted with the user’s message, while model responses can stream into the chat interface as text. Capture-bridge and request-packaging details are provided in Supplementary Chapter S3.

### Benchmark evaluation

Two benchmark presets were run with the offscreen capture pipeline at 1,000 × 1,000 pixels.

#### Determinism preset

Ten two-dimensional viewer states from one series were each captured five times (50 frames total) with overlays disabled. A warm-up pass executed each unique state once without logging. Repeatability was assessed by Secure Hash Algorithm 256 hashing of the captured pixel buffer and comparison of repeated captures of the same serialized state (Supplementary Chapter S2).

#### Mixed performance preset

A batch of two-dimensional and multiplanar reconstruction frames (axial, coronal, sagittal) was captured with overlays enabled. The first frame was omitted to reduce cold-start effects, leaving 39 logged frames. Per-frame capture timing, JPEG and HEIC encoding time, and output size were recorded; capture time denotes wall-clock duration from capture initiation to completion callback, with encoding reported separately (Supplementary Chapter S2).

### Benchmark environment and statistical analysis

Evaluation was performed on an M2 MacBook Air with macOS 15.5 using the application’s iOS UIKit target, Visualization Toolkit 9.3.20240830, and OpenGL for Embedded Systems 3.0. Test data were one axial magnetic resonance imaging (MRI) series (512 × 384 matrix, 192 slices, 0.488 × 0.488 mm spacing, 1-mm thickness), taken from the UPenn-GBM collection *via* The Cancer Imaging Archive [8, 9]. Reported latency results reflect hot-cache capture with the source series resident in shared memory; performance may differ for larger datasets, other modalities, or cold-cache and networked environments. Logs were summarized in Python, with medians and interquartile ranges reported by view type. Environment details, logged fields, and summary rules are provided in Supplementary Chapter S2.

### Use of large language models

Writing assistance for this manuscript was provided in part by large language models (gpt-5.2-high (OpenAI Inc.) and Gemini 3 Pro Preview (Google LLC)); the authors remain solely responsible for its content.

## Results

### Evidence export types

The viewer supports four evidence types aligned to clinical intent: current-view capture, sampled slice stacks, orthogonal MPR capture, and annotated bookmark capture. A summary of evidence types and preserved viewing context is provided in Supplementary Chapter S3. Captures preserve view-defining parameters through explicit state descriptors rather than pixel scraping. For each exported frame, the system can associate a replayable viewer state (series, mode, slice/plane intent, window/level, slab settings, and overlay policy), enabling later reproduction and audit of the exact evidence provided to the model.

### Evidence staging workflow

**Fig. 2:**
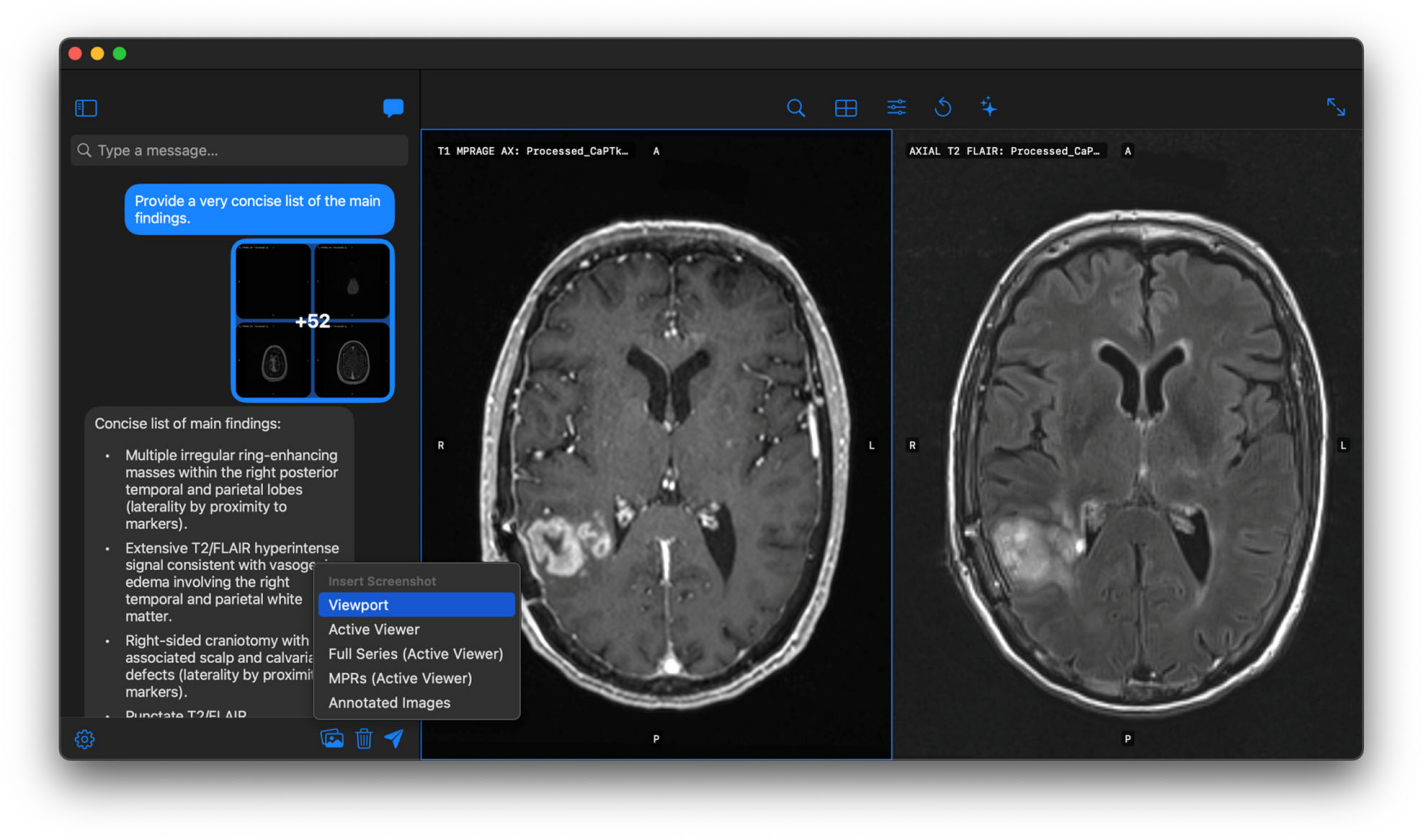
The chat capture interface. Key workflow steps: (1) the user selects a capture action from the sidebar (eg, current view, sampled slice stack, orthogonal multiplanar reconstruction, annotated bookmarks); (2) the system generates a batch of viewer states and renders them offscreen *via* an apply-and-capture loop that yields between frames to keep the diagnostic grid responsive; (3) thumbnails appear progressively as frames complete (media accumulation), allowing the user to draft questions while evidence is still assembling; (4) the user reviews staged evidence and sends the message. Images are not transmitted until submission. The capture loop advances *via* completion callbacks (with timeout fallback), so processing continues even if a state application stalls. Imaging shown is sourced from the UPenn-GBM collection *via* The Cancer Imaging Archive refs. [8, 9]. GBM, Glioblastoma multiforme; UPenn, University of Pennsylvania

**Fig. 3:**
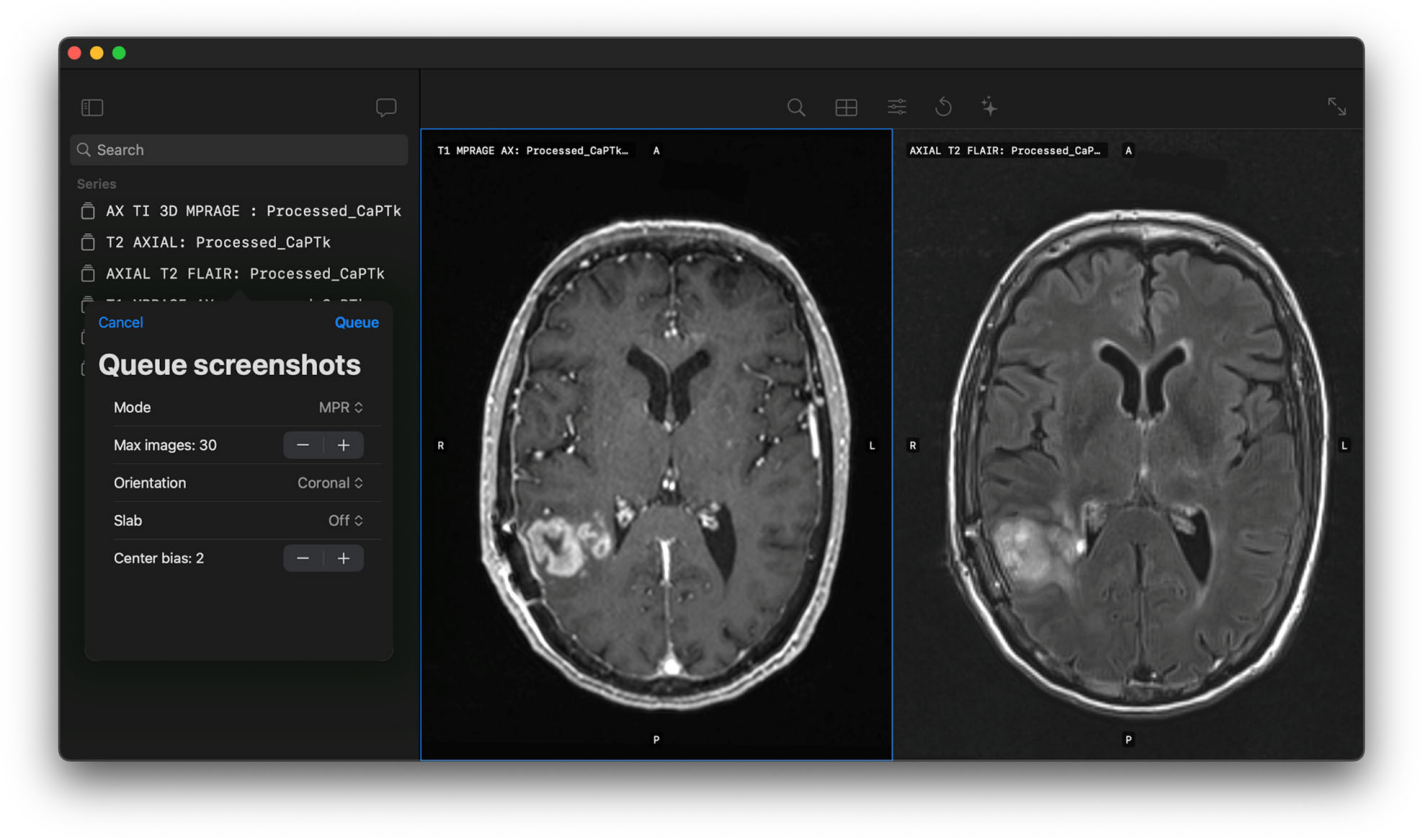
Batch queuing from navigation. Key workflow steps: (1) the user selects a series from the navigation list without loading it into the diagnostic grid; (2) viewer states are constructed algorithmically from capture presets (*e.g*., max frame count, sampling strategy, plane intent, slab mode/thickness, window/level defaults); (3) the offscreen capture instance renders frames using shared cached voxel data without disrupting interactive viewing; (4) staged evidence is available for later message composition (“stage now, ask later”). The capture loop advances *via* callbacks with timeout handling. Each exported frame corresponds to an explicit logged state, supporting repeatable evidence generation. Imaging shown is sourced from the UPenn-GBM collection *via* The Cancer Imaging Archive refs. [8, 9]. GBM, Glioblastoma multiforme; UPenn, University of Pennsylvania

The sidebar exposes capture actions corresponding to the evidence types described above (Figs. 2 and 3). Because capture may produce multiple frames, exported images are staged in the sidebar prior to transmission so users can curate evidence attached to a message. The interface supports incremental “media accumulation,” showing placeholders immediately and replacing them with thumbnails as frames complete, allowing question drafting while export proceeds. Batch execution advances through the queued states without blocking interactive viewing, and evidence is transmitted only when the user sends the message (Supplementary Chapter S3).

### Determinism

In the determinism benchmark (10 two-dimensional states, five repeats per state), no final-hash mismatches were observed (0 of 50 frames). Hashing was performed over the captured pixel buffer at a fixed output resolution (1,000 × 1,000 pixels), with overlays disabled to isolate repeatability within the run under identical serialized state. Per-state summaries reported zero mismatches for all 10 states (Supplementary Chapter S2). These results demonstrate deterministic output under the controlled benchmark conditions described (single device, hot cache, single MRI series, single execution run); reproducibility across heterogeneous deployment environments was not assessed.

### Capture performance

In the mixed performance benchmark (39 logged frames; 1,000 × 1,000 pixel output; overlays enabled), median capture times differed by view type (Table 1). Two-dimensional frames and axial MPR were ms-scale in the logged batch, with wider interquartile ranges (two-dimensional: median 6.19 ms [4.69, 9.73]; axial MPR: median 4.49 ms [3.97, 10.73]) than coronal and sagittal MPR. Coronal and sagittal MPR capture times were longer but more tightly distributed (medians 16.55 ms and 16.69 ms, respectively). The longer capture times for coronal and sagittal MPR are consistent with the computational cost of orthogonal reslicing: the test series was acquired in the axial plane (in-plane matrix 512 × 384, 192 slices), so axial MPR can read contiguous in-plane data, whereas coronal and sagittal reslicing requires interpolation across non-contiguous memory locations, increasing computation time.

**Table 1:** Benchmark summary for determinism, capture latency, and encoding tradeoffs

Metric category	View or format	Logged frames (*n*)	Output resolution (pixels)	Overlays	Value (median [interquartile range])
Capture time, ms	Two-dimensional	9	1,000 × 1,000	Enabled	6.19 [4.69, 9.73]
Capture time, ms	MPR, axial	10	1,000 × 1,000	Enabled	4.49 [3.97, 10.73]
Capture time, ms	MPR, coronal	10	1,000 × 1,000	Enabled	16.55 [16.31, 16.70]
Capture time, ms	MPR, sagittal	10	1,000 × 1,000	Enabled	16.69 [16.21, 16.87]
Encoding time, ms	JPEG (quality 0.7)	39	-	-	3.66 [2.73, 4.04]
File size, kilobytes	JPEG (quality 0.7)	39	-	-	79.4 [65.8, 92.1]
Encoding time, ms	HEIC (quality 0.7)	39	-	-	28.43 [28.06, 29.08]
File size, kilobytes	HEIC (quality 0.7)	39	-	-	27.9 [23.3, 33.5]

The first frame of the mixed batch was omitted from logging (40 frames captured; 39 logged). Determinism preset result: 0 mismatches among 50 frames (10 states × 5 repeats). Encoding metrics were measured on a background serial queue; additional end-to-end fields are logged in per-frame records (Supplement S2). Kilobyte values are computed from bytes using 1,000 bytes per kilobyte

*HEIC* High-efficiency image container, *JPEG* Joint photographic experts group, MPR Multiplanar reconstruction

### Encoding tradeoffs

At the chosen encoder setting (quality 0.7), HEIC encoding produced smaller files but required longer encoding time than JPEG encoding (Table 1). Median HEIC output size was 27.9 kilobytes *versus* 79.4 kilobytes for JPEG (approximately 2.8× smaller), while median HEIC encoding time was 28.43 ms *versus* 3.66 ms for JPEG (approximately 7.8× longer).

## Discussion

This work describes a radiology viewer architecture that enables in-viewer vision-language model (VLM) interactions with minimal workflow disruption, avoiding the reduction of volumetric interpretation to *ad hoc* screenshots or external export-and-upload steps. The core mechanism is explicit serialization of viewing state, allowing an offscreen viewer instance to re-render model-ready two-dimensional evidence while preserving clinically meaningful context, including plane intent, slice selection, window/level, slab configuration, and overlay policy. A dedicated assistant sidebar supports evidence staging and progressive thumbnail aggregation while keeping the diagnostic grid uninterrupted.

This architecture addresses a gap identified by recent work on multimodal artificial intelligence in radiology. Evaluations of multimodal foundation models have underscored the importance of reliable alignment between image content and its clinical viewing context during model interaction [10]. Studies of vision-enabled large language models further demonstrate that these systems typically operate on exported two-dimensional images rather than native DICOM data, highlighting the need for context-preserving export mechanisms within diagnostic viewers [11]. Related work on workflow-integrated artificial intelligence in radiology emphasizes the value of embedding model interaction directly within the clinical environment, rather than treating it as a separate, downstream step [12].

The proposed architecture treats model interaction as an extension of the diagnostic viewing process rather than a separate downstream operation. Diagnostic reasoning in radiology rarely depends on a single frame in isolation, instead incorporating spatial continuity across slice batches, multiplanar context, and correlations between multiple series or prior studies. By serializing explicit viewing state and supporting coordinated multi-frame capture across related viewing contexts, the system preserves a richer representation of diagnostic intent while enabling reproducible and auditable model interaction.

Benchmarks demonstrated pixel-identical exports across repeated replays within a single run under fixed viewing state and output resolution, with no final-hash mismatches observed across repeated captures. Capture latency was millisecond-scale for two-dimensional views and MPR in the logged batches, with longer capture times observed for coronal and sagittal MPR views. Encoding experiments revealed a size–latency trade-off: HEIC encoding substantially reduced output size but incurred higher encoding latency compared with JPEG format. From a clinical workflow perspective, HEIC’s smaller file size may be advantageous when submitting multiple frames over bandwidth-constrained connections or when downstream model endpoints impose request size limits, whereas JPEG’s faster encoding may be preferable for time-sensitive single-frame interactions.

The design supports traceability by ensuring that each exported frame corresponds to an explicit, replayable viewer state that can be logged, audited, and reviewed in deployment. Optional overlay burn-in enables preservation of clinically meaningful annotations, markers, and measurements; however, overlay content must be carefully governed to prevent unintended exposure of protected health information.

This architecture suggests two practical directions for future development. The first is intent-driven capture templates, in which common clinical questions are paired with standardized capture configurations, such as plane presets, slice sampling strategies, window/level defaults, and slab thickness, along with corresponding prompt framing; over time, these templates could evolve into versioned, replayable “context bundles” that institutions can standardize, validate, and evaluate for consistency and effectiveness. The second is bidirectional interaction: when models return structured outputs, such as candidate regions of interest or prioritized views, the same serialized-state mechanism could be used to render these results back into the viewer as optional overlays aligned with the exported evidence; this approach would support verification, interpretability, and explainability within the radiologist’s normal diagnostic viewing context.

Several limitations should be noted. Benchmarks were conducted on a single device under hot-cache conditions using one MRI series and a single logged run; performance and repeatability should therefore be validated across imaging modalities, datasets, devices, and cold-cache or networked environments to better characterize tail latency and operational variability. The architecture relies on a shared in-memory voxel cache that retains per-series volumetric data for all loaded studies; when multiple large volumetric datasets are opened concurrently, such as multiphase computed tomography or multisequence MRI examinations, aggregate memory usage may become substantial, necessitating cache eviction strategies or explicit memory budgets to ensure stability on resource-constrained systems. Finally, additional evaluation is required to assess the impact of integrated evidence capture and in-viewer model interaction on radiologist workflow, including effects on efficiency, reliability, safety, and user trust under realistic clinical reading conditions.

## Supplementary information

**Additional File: Fig. S1** Viewport layering. Each grid cell is a wrapper that coordinates (1) a high-performance rendering layer (VTK/OpenGL pipeline for two-dimensional slice and multiplanar reconstruction rendering) and (2) a vector overlay layer for orientation markers, labels, and measurement graphics. The renderer draws from a shared in-memory per-series voxel cache so multiple viewports and the offscreen capture instance can reuse the same volume data without duplicating large buffers, while each viewer maintains independent visualization state (slice selection, window/level, slab settings, camera). Separating overlays from the voxel renderer preserves overlay sharpness across zoom and resolution changes and enables policy-controlled export: overlays can be excluded (for repeatability tests or protected health information controls) or burned into exported pixels when they carry clinical meaning (for example, measurements and laterality markers). OpenGL Open graphics library, VTK Visualization toolkit. **Fig. S2** Rendering and capture bridge. Offscreen export uses GPU-to-GPU copying into a pixel-buffer-backed target to avoid synchronous glReadPixels stalls. The renderer renders into its source framebuffer and then blits (glBlitFramebuffer) into a snapshot framebuffer object (FBO) whose color attachment is a texture view of an IOSurface-backed pixel buffer (BGRA8 pixel format) created via a texture cache. The resulting capture buffer can be hashed (eg, SHA-256 over BGRA8 rows) for repeatability evaluation and passed downstream for optional overlay compositing and image encoding. Benchmark-only GPU synchronization (eg, glFinish) can be enabled to bound timing variability when measuring component latencies. BGRA8 Blue-Green-Red-Alpha, 8-bit per channel pixel format, FBO Framebuffer object, GPU Graphics processing unit, SHA-256 Secure hash algorithm 256. **Fig. S3** Compositing and packaging. The captured pixel buffer is locked for CPU access during snapshot finalization and optionally composited with an overlay layer (e.g., measurements, labels, and orientation markers) by drawing into a bitmap context backed directly by the pixel-buffer memory, producing a single fused frame. Frames are then encoded as JPEG or HEIC and staged locally before submission. The chat request is sent as an HTTP POST with the staged attachments, while model responses stream back into the transcript via server-sent events, separating local staging from remote inference latency. CPU Central processing unit, HEIC High-efficiency image container, HTTP Hypertext transfer protocol, JPEG Joint photographic experts group, POST HTTP POST method. **Table S1** Logged benchmarking metric definitions (key and additional fields). **Table S2** Determinism per-state summary rows (series identifier redacted). **Table S3** Evidence types and preserved viewing context for model-ready export.

**Video 1:** In-viewer vision-language model workflow with state-based evidence export. The application initially shows the assistant sidebar in Chat mode (empty transcript) alongside a 1×2 diagnostic grid displaying two axial brain MRI series (left: T1-weighted post-contrast; right: T2-weighted). The user switches the sidebar to Navigation mode and schedules a capture batch directly from the series list: orthogonal MPRs (axial/coronal/sagittal) for the T1 post-contrast series and a native 2D axial slice batch (non-MPR) for the T2 series. After confirming enqueue, the system generates model-ready images via offscreen re-rendering from serialized viewing state and returns to Chat mode, showing progressive thumbnail accumulation as captures complete. The user then submits the prompt (“describe the findings concisely”) together with the staged images; after upload and remote inference, the model’s answer is streamed into the chat transcript. Imaging is sourced from the UPenn-GBM dataset (TCIA).

## Data Availability

Relevant code is available at: https://github.com/radiodiagnostica/vlm-vtk-uikit-resources.

## Abbreviations

DICOM: Digital Imaging and Communications in Medicine
HEIC: High-efficiency image container
JPEG: Joint photographic experts group
MPR: Multiplanar reconstruction
MRI: Magnetic resonance imaging
VLM: Vision-language model
