# Supplementary material for: Technical architecture for integrating vision-language models with DICOM viewers
Source: Eur Radiol Exp. 2026 Jul 1;10:103. doi: 10.1186/s41747-026-00769-0 (PMC13323766; doi:10.1186/s41747-026-00769-0)
Supplement: Supplementary file 1 — Additional File: Fig. S1 Viewport layering. Each grid cell is a wrapper that coordinates (1) a high-performance rendering layer (VTK/OpenGL pipeline for two-dimensional slice and multiplanar reconstruction rendering) and (2) a vector overlay layer for orientation markers, labels, and measurement graphics. The renderer draws from a shared in-memory per-series voxel cache so multiple viewports and the offscreen capture instance can reuse the same volume data without duplicating large buffers, while each viewer maintains independent visualization state (slice selection, window/level, slab settings, camera). Separating overlays from the voxel renderer preserves overlay sharpness across zoom and resolution changes and enables policy-controlled export: overlays can be excluded (for repeatability tests or protected health information controls) or burned into exported pixels when they carry clinical meaning (for example, measurements and laterality markers). OpenGL Open graphics library, VTK Visualization toolkit. Fig. S2 Rendering and capture bridge. Offscreen export uses GPU-to-GPU copying into a pixel-buffer-backed target to avoid synchronous glReadPixels stalls. The renderer renders into its source framebuffer and then blits (glBlitFramebuffer) into a snapshot framebuffer object (FBO) whose color attachment is a texture view of an IOSurface-backed pixel buffer (BGRA8 pixel format) created via a texture cache. The resulting capture buffer can be hashed (eg, SHA-256 over BGRA8 rows) for repeatability evaluation and passed downstream for optional overlay compositing and image encoding. Benchmark-only GPU synchronization (eg, glFinish) can be enabled to bound timing variability when measuring component latencies. BGRA8 Blue-Green-Red-Alpha, 8-bit per channel pixel format, FBO Framebuffer object, GPU Graphics processing unit, SHA-256 Secure hash algorithm 256. Fig. S3 Compositing and packaging. The captured pixel buffer is locked for CPU access during snapshot finalization a [file 41747_2026_769_MOESM1_ESM.pdf]

Technical architecture for integrating vision-language models with DICOM viewers

ELECTRONIC SUPPLEMENTARY MATERIAL

Abbreviations

- **BGRA8**: Blue-Green-Red-Alpha, 8-bit per channel pixel format
- **CPU**: Central processing unit
- **CSV**: Comma-separated values
- **DICOM**: Digital Imaging and Communications in Medicine
- **FBO**: Framebuffer object
- **GPU**: Graphics processing unit
- **HEIC**: High Efficiency Image Container
- **HTTP**: Hypertext Transfer Protocol
- **I/O**: Input/output
- **JPEG**: Joint Photographic Experts Group
- **JSON**: JavaScript Object Notation
- **JSONL**: JSON Lines
- **MinIP**: Minimum intensity projection
- **MIP**: Maximum intensity projection
- **MPR**: Multiplanar reconstruction
- **OpenGL**: Open Graphics Library
- **OpenGL ES**: OpenGL for Embedded Systems
- **POST**: HTTP POST method
- **pt**: Points (typographic/display points)
- **px**: Pixels
- **ROI**: Region of interest
- **SHA-256**: Secure Hash Algorithm 256
- **SSE**: Server-sent events
- **UID**: Unique identifier
- **UI**: User interface
- **VTK**: Visualization Toolkit

Supplementary Chapter S1. Viewer state descriptor overview

The fields required to reproduce exported evidence from saved state are summarized in the following subchapters.

S1.1 Core state fields

- **Series identifier:** identifies the selected series for rendering (for example, DICOM Series Instance UID or an internal stable identifier).
- **Rendering mode:** two-dimensional or multiplanar reconstruction (MPR).
- **Plane intent and slice selection:**
    - two-dimensional: slice_index (integer), optional slice_count (for stacks);
    - MPR: orientation_intent (axial/coronal/sagittal) and a position in the reslice axis.
- **Window and level:** intensity display mapping parameters, typically {window, level}.
- **Slab configuration:** slab mode and slab thickness when enabled, for example:
    - slab.mode (∈ {none, mip, minip, average})
    - slab.thickness_mm (float)
- **Camera geometry (when applicable):** parameters sufficient to restore camera configuration repeatably for the relevant rendering mode. Example fields (names illustrative):
    - camera.position (3-vector)
    - camera.focal_point (3-vector)
    - camera.view_up (3-vector)
    - camera.parallel_scale (float) for parallel projection workflows
    - optional camera.clipping_range (2-vector)
- **Overlay policy:** whether overlays are excluded or burned into exported pixels, for example:
    - overlay.include = false | true
    - optional fine-grained toggles for label classes (orientation marker, rulers, regions of interest, text labels)

S1.2 Batch state generation

Batch capture actions generate an ordered sequence of viewer states that vary in slice selection and/or plane intent while keeping other parameters fixed, matching the user's capture intent (for example, a sampled stack in the current plane or an orthogonal MPR set). For slice-stack capture, the goal is to select slice indices from a volume with N slices to produce up to K exported frames.

Uniform sampling

Uniform sampling selects indices by partitioning the slice range into K bins and taking the left edge of each bin, with the final index set to the last slice. Let:

$$K = \min(\text{maxEntries}, N).$$

For $j = 0, \dots, K-1$, define:

$$i_j = \begin{cases} N-1, & j = K-1, \\ \left\lfloor \frac{j\,N}{K} \right\rfloor, & 0 \le j < K-1. \end{cases}$$

Center-biased sampling

Center-biased sampling allocates denser sampling near the volume center while preserving peripheral coverage. It uses a symmetric power transform around the midpoint with exponent $p > 1$. For $j = 0, \dots, K-1$, define the normalized position:

$$u = \begin{cases} 1, & j = K-1, \\ \frac{j}{K-1}, & 0 \le j < K-1. \end{cases}$$

$$t = 2u - 1 \quad (\text{so } t \in [-1,1]),$$

$$v = \frac{1}{2} + \frac{1}{2}\,\operatorname{sgn}(t)\,|t|^p \quad (\text{so } v \in [0,1]),$$

and map to a slice index:

$$i_j = \left\lfloor v\,(N-1) + \frac{1}{2} \right\rfloor.$$

Unless otherwise specified, $p = 2$ was used as the default center-bias exponent.

Post-processing rules

After index generation, indices are clamped to the valid range $0 \le i_j \le N-1$, de-duplicated if rounding collisions occur, and retained in ascending order. When $K \ge 2$, the first and last indices (0 and $N-1$) are ensured to be included to preserve end-to-end coverage.

Supplementary chapter S2. Benchmark configuration and additional results detail

S2.1 Benchmark presets

- **Determinism preset:** 10 two-dimensional states; five repeats per state; overlays disabled; pixel hashing enabled; warm-up pass executed but not logged.
- **Mixed performance preset:** two-dimensional plus MPR frames (axial, coronal, sagittal); overlays enabled; first frame omitted from logging; 39 frames logged (two-dimensional, n = 9; axial/coronal/sagittal MPR, n = 10 each); per-frame timing and encoding metrics logged. JPEG and HEIC encoding at quality 0.7 was performed on a background serial queue.

All benchmark frames were rendered at 1000 × 1000 px.

S2.2 Benchmark environment

Benchmarks were run as a single-device evaluation on an M2 MacBook Air (macOS 15.5 [24F74]) using the application's iOS UIKit target (non-Catalyst), VTK 9.3.20240830, and OpenGL ES 3.0. Test imaging data consisted of a single axial MRI series (in-plane matrix 512 × 384 [rows × columns], 192 slices; pixel spacing 0.488 × 0.488 mm; slice thickness 1.0 mm). For the reported capture-latency results, the source series was already loaded in the shared in-memory voxel cache at capture time (hot-cache condition). Performance may differ for larger datasets, other imaging modalities (*e.g.*, computed tomography), or networked environments where cold-cache loading dominates.

S2.3 Warm-up and cold-start handling

In determinism mode, a warm-up pass executes each unique state once without logging prior to the repeated captures. In performance mode, the first frame of the batch is omitted from logging to reduce cold-start bias, while still producing the frame.

S2.4 Logged outputs and formats

Benchmark logs are emitted in line-delimited JSON (JSONL) and CSV for analysis.

S2.5 Logged fields (high level)

Per exported frame, logs include:

- identifiers: run_id, index, series_uid, state_id, repeat, and capture_mode,
- output size: w_px, h_px, and bytes_per_row,
- capture timing: t_apply_state_ms, t_capture_total_ms, and component timings emitted by the capture pipeline,

- optional encoding metrics: JPEG and HEIC encoding times and sizes at quality 0.7, and derived end-to-end times.

S2.6 Logged metric definitions (for reproducibility and debugging)

The manuscript's quantitative summaries are based on t_capture_total_ms, pixel-hash determinism fields, and the encoding time/size fields; other component timings were logged for reproducibility and debugging and are provided for completeness. Logged benchmarking metric definitions are summarized in Supplementary Table S1.

Table S1 here.

S2.7 Pixel hash definition

Pixel hashes are computed using Secure Hash Algorithm 256 (SHA-256) over the pixel buffer base address for the rendered frame. The hash function iterates over rows and hashes exactly width × 4 bytes per row (BGRA8), skipping any per-row padding beyond that (stride-aware via bytes_per_row). In determinism benchmarking, overlays are disabled; hashes therefore reflect repeatability within the run under identical viewing state and fixed output size.

S2.8 Determinism state summaries (redacted identifiers)

The determinism run produced 10 per-state summary rows. Series identifiers are redacted; slice indices are preserved. Determinism per-state summary rows are shown in Supplementary Table S2.

Table S2 here.

S2.9 Summary computation note

Summary statistics reported in the manuscript are computed over the logged frames as medians and interquartile ranges (25th–75th percentiles). No frames were excluded from summaries beyond the preset's predefined logging rules (for example, omission of the first frame in the mixed performance preset).

Supplementary chapter S3. Additional implementation notes for capture, scheduling, and overlays

S3.0 Workflow terminology

Evidence capture can be initiated from an active diagnostic viewport or directly from a series list. Capture does not screenshot displayed pixels; instead, each request is translated into one or more explicit viewer-state descriptors. Those states are executed by a separate offscreen viewer instance that reuses in-memory imaging data and returns exported frames to the sidebar for selection prior to submission to a model endpoint.

In this manuscript, *capture* denotes offscreen rendering and pixel extraction of a frame from a serialized viewing state; *export* denotes encoding a captured frame into a transmissible image format; and *evidence* denotes the curated set of exported images staged for model submission.

S3.1 Shared imaging data, viewing modes, and viewer-instance state

Supported viewing modes include two-dimensional slice navigation and axial, coronal, and sagittal multiplanar reconstruction (MPR), with optional slab rendering and configurable thickness. Overlays (*e.g.*, orientation markers and measurement graphics) are drawn above the rendered image in a separate overlay layer. For export, overlays are controlled by policy and can be excluded or composited into the exported pixels.

To reduce I/O and memory duplication, per-series slice and volume objects (for example, vtkImageData) are retained in a shared cache keyed by series identity and reused by interactive viewports and the offscreen capture instance. Viewer instances maintain independent visualization configuration (rendering mode, slice selection, window and level, slab, camera, overlay policy), enabling concurrent interactive viewing and background export without duplicating voxel buffers. Viewport layering is illustrated in Supplementary Fig. S1.

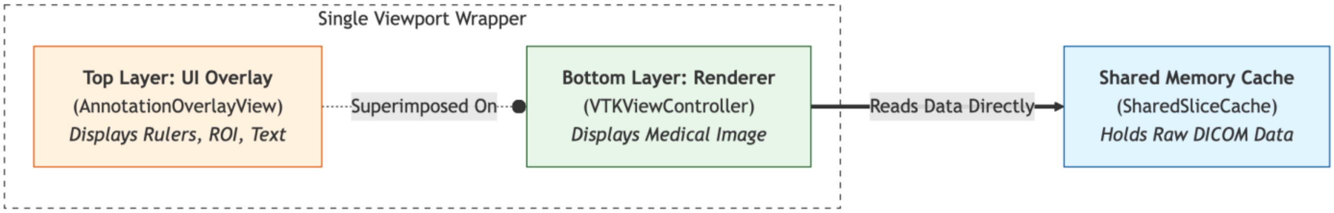

Fig. S1 Viewport layering. Each grid cell is a wrapper that coordinates (1) a high-performance rendering layer (VTK/OpenGL pipeline for two-dimensional slice and multiplanar reconstruction rendering) and (2) a vector overlay layer for orientation markers, labels, and measurement graphics. The renderer draws from a shared in-memory per-series voxel cache so multiple viewports and the offscreen capture instance can reuse the same volume data without duplicating large buffers, while each viewer maintains independent visualization state (slice selection, window/level, slab settings, camera). Separating overlays from the voxel renderer preserves overlay sharpness across zoom and resolution changes and enables policy-controlled export: overlays can be excluded (for repeatability tests or protected health information controls) or burned into exported pixels when they carry clinical meaning (for example, measurements and laterality markers). *OpenGL* Open graphics library, *VTK* Visualization toolkit.

S3.2 Asynchronous loading and request de-duplication

To avoid redundant I/O, series/volume loading is mediated by an asynchronous fetch manager that de-duplicates concurrent requests for the same underlying data. When multiple consumers request the same series (for example, multiple live viewports plus an offscreen capture batch), the system ensures the load occurs once and that all consumers receive completion callbacks when the data becomes available. Request prioritization can favor interactive viewing over background capture.

S3.3 Apply-and-capture scheduling and timeout handling

On the target UI stack, view hierarchy manipulation and rendering coordination are main-thread-bound. Responsiveness is preserved by a cooperative apply-and-capture loop: for each queued viewer state, the pipeline applies state, yields to allow UI/layout/render commits, captures the frame, then advances to the next state. The loop is callback-driven (apply completion → capture completion → next item), with timeout handling to advance the queue if state application does not complete within a configured bound (see timeout in Supplementary Chapter S2).

S3.4 Evidence staging and incremental UI updates

Because capture actions may produce multiple frames, the sidebar stages exported evidence locally prior to transmission. The UI can insert placeholders immediately and replace them with thumbnails as frames complete, allowing the user to continue interacting with the diagnostic grid and drafting text while background export proceeds. Evidence is transmitted only when the user sends a message. Evidence types and preserved viewing context are summarized in Supplementary Table S3.

Table S3 here.

S3.5 Capture target, offscreen host, GPU/CPU boundaries, and compositing

Captured frames are produced by an offscreen viewer instance driven by explicit viewer state. In the current implementation, the offscreen instance is created by cloning the same viewer/view hierarchy used for interactive rendering and attaching it to an offscreen "host" container (a staging window/view) that can be deterministically sized to a configured export dimension (fixed in points; pixel dimensions are points × device scale, *e.g.*, 500×500 pt at 2× yields 1000×1000 px). This avoids dependence on the on-screen diagnostic viewport and keeps export behavior consistent across interactive layout changes. Export resolution refers to the offscreen viewport size; image content is rendered without geometric stretching (fit-to-view is implemented via camera scaling), and any aspect mismatch appears as background padding within the exported frame.

For each queued viewer state, the pipeline (1) applies the state to the offscreen viewer, (2) renders into the renderer's framebuffer, and (3) copies/blits the rendered result into a pixel-buffer-backed capture target. The resulting pixel buffer serves as the interchange point between the GPU rendering stage and downstream steps: (a) hashing for repeatability evaluation, (b) optional overlay compositing, and (c) image encoding for model ingestion. Frames are encoded in the display orientation produced by the offscreen viewer render.

When overlays are enabled for export, the pixel buffer is composited with the overlay layer (for example, orientation markers and measurement graphics) to produce a single fused frame. The fused frame is then converted into a platform image object for encoding (JPEG or HEIC) and local staging. Low-level capture and compositing mechanics are illustrated in Supplementary Figs. S2 and S3.

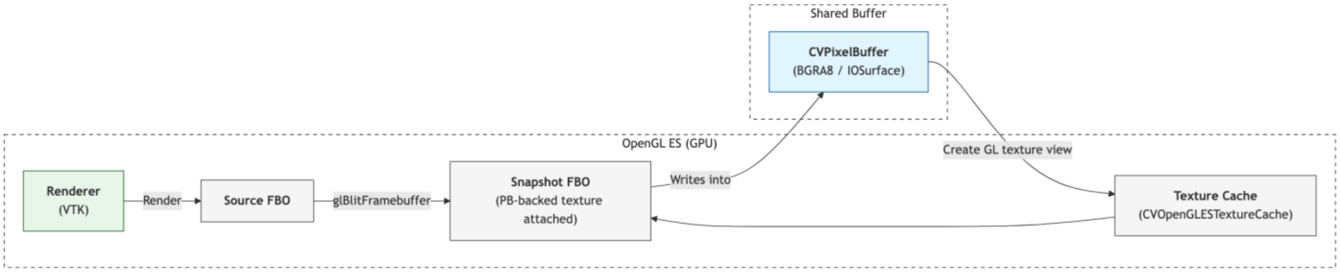

Fig. S2 Rendering and capture bridge. Offscreen export uses GPU-to-GPU copying into a pixel-buffer-backed target to avoid synchronous glReadPixels stalls. The renderer renders into its source framebuffer and then blits (glBlitFramebuffer) into a snapshot framebuffer object (FBO) whose color attachment is a texture view of an IOSurface-backed pixel buffer (BGRA8 pixel format) created via a texture cache. The resulting capture buffer can be hashed (eg, SHA-256 over BGRA8 rows) for repeatability evaluation and passed downstream for optional overlay compositing and image encoding. Benchmark-only GPU synchronization (eg, glFinish) can be enabled to bound timing variability when measuring component latencies. *BGRA8* Blue-Green-Red-Alpha, 8-bit per channel pixel format, *FBO* Framebuffer object, *GPU* Graphics processing unit, *SHA-256* Secure hash algorithm 256.

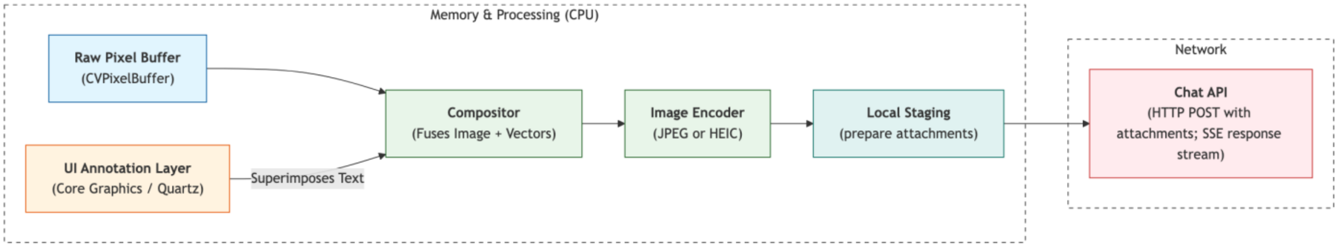

Fig. S3 Compositing and packaging. The captured pixel buffer is locked for CPU access during snapshot finalization and optionally composited with an overlay layer (e.g., measurements, labels, and orientation markers) by drawing into a bitmap context backed directly by the pixel-buffer memory, producing a single fused frame. Frames are then encoded as JPEG or HEIC and staged locally before submission. The chat request is sent as an HTTP POST with the staged attachments, while model responses stream back into the transcript via server-sent events, separating local staging from remote inference latency. CPU Central processing unit, HEIC High-efficiency image container, HTTP Hypertext transfer protocol, JPEG Joint photographic experts group, POST HTTP POST method.

S3.6 Overlay export policy and protected health information considerations

Overlay export is treated as a configurable policy because overlays may include identifying text depending on deployment configuration. Clinically meaningful overlays may include measurements or laterality markers. Export pipelines must ensure that identifiers do not appear in exported pixels or filenames. Implementations may also support finer-grained controls (*e.g.*, include measurements but exclude free-text labels) depending on institutional policy. The current implementation does not perform automated burned-in-text detection or optical character recognition-based filtering; prevention relies on deployment-level overlay configuration and institutional policy.

S3.7 Request packaging and streaming responses

Exported images are attached to a chat request only after local staging and user confirmation. Model integration is isolated behind a provider adapter that packages selected images and text into the provider's request schema. Responses can be rendered as streaming text in the transcript (*e.g.*, Server-Sent Events-style incremental tokens), decoupling local evidence assembly latency from remote inference latency.

Supplementary Tables

Table S1 Logged benchmarking metric definitions (key and additional fields)

Field	Definition (as logged)
t_resize_ms	Time to resize/layout the offscreen host window/view hierarchy to the benchmark fixed size for the current item.
t_apply_state_ms	Time from initiating viewer-state application to the apply completion callback (state applied; render is not forced in the apply call).
t_capture_total_ms	Wall-clock time from initiating capture (captureScreenshot(...)) to its completion callback, which returns the exported UIImage and optional metrics.
t_vtk_render_ms	Renderer-reported time for the VTK render step for the captured frame (as emitted by the rendering controller).

Field	Definition (as logged)
t_blit_ms	Time to issue the framebuffer blit into the capture target (FBO setup + glBlitFramebuffer + glFlush); GPU completion may occur later unless explicit synchronization is enabled.
t_gpu_sync_ms	Time spent in an explicit GPU synchronization step when enabled (benchmark-only; glFinish).
t_lock_ms	Time to lock the CVPixelBuffer base address for CPU access; may include implicit synchronization waiting for prior GPU writes to the IOSurface-backed buffer when explicit GPU sync is not used.
t_overlay_ms	Time to draw overlays into the export buffer when overlays are enabled.
overlay_items	Number of overlay elements drawn into the export buffer when overlays are enabled.
t_cgimage_ms	Time to create a CGImage from the bitmap context wrapping the pixel buffer memory.
t_uiimage_ms	Time to construct a UIImage wrapper from the CGImage.
hash_gl	SHA-256 hash computed over the pixel buffer bytes prior to overlay drawing (stride-aware; BGRA8).
hash_final	SHA-256 hash computed over the pixel buffer bytes after overlay drawing (stride-aware; BGRA8).
hash_mismatch	Determinism-only flag indicating whether hash_final differs from the baseline repeat for the same state_id.
enc_sampled	Whether encoding was performed for the current frame under the sampling policy.
enc_quality	Lossy encoding quality used for the encoding benchmark (0–1 scale).

Field	Definition (as logged)
enc_every_n	Encoding sampling policy; encode every Nth frame.
enc_jpeg_q70_ms, enc_jpeg_q70_bytes	JPEG encoding time and output size at the configured quality.
enc_heic_q70_ms, enc_heic_q70_bytes	HEIC encoding time and output size at the configured quality.
t_e2e_jpeg_q70_ms, t_e2e_heic_q70_ms	Derived end-to-end time computed as t_capture_total_ms + enc_*_ms when encoding is sampled.
t_e2e_with_apply_jpeg_q70_ms, t_e2e_with_apply_heic_q70_ms	Derived end-to-end time computed as t_apply_state_ms + t_capture_total_ms + enc_*_ms when encoding is sampled.
timeout	Boolean indicating the state application timed out and the pipeline advanced to the next item.

BGRA8, Blue-Green-Red-Alpha, 8-bit per channel pixel format; FBO, framebuffer object; GPU, graphics processing unit; HEIC, High Efficiency Image Container; JPEG, Joint Photographic Experts Group; SHA-256, Secure Hash Algorithm 256; VTK, Visualization Toolkit.

Table S2 Determinism per-state summary rows (series identifier redacted)

State (redacted)	Frames per state	Mismatches
<series>_2d_slice0	5	0
<series>_2d_slice19	5	0
<series>_2d_slice38	5	0
<series>_2d_slice57	5	0
<series>_2d_slice76	5	0
<series>_2d_slice96	5	0
<series>_2d_slice115	5	0
<series>_2d_slice134	5	0
<series>_2d_slice153	5	0
<series>_2d_slice191	5	0

Table S3 Evidence types and preserved viewing context for model-ready export

Evidence type	Exported frames	Preserved context (examples)	Overlays in export	Typical use
Current view capture	1	Active series, view type, plane, slice position, window and level, slab configuration, camera geometry when applicable	Optional	"Show the current finding"
Sampled slice stack	Up to N	Series identity, ordered slice sampling strategy, window and level, plane, and slice indices	Optional	"Provide representative volumetric context"
Orthogonal MPR	Up to N per plane	Plane intent, slab type and thickness, window and level	Optional	"Show orthogonal context around a finding"
Annotated bookmark capture	1 per bookmark	Bookmark identity, associated series and viewing state, optional measurement overlays	Optional (often enabled)	"Show the marked finding and intent"

MPR Multiplanar reconstruction.

Video 1: In-viewer vision-language model workflow with state-based evidence export. The application initially shows the assistant sidebar in Chat mode (empty transcript) alongside a 1×2 diagnostic grid displaying two axial brain MRI series (left: T1-weighted post-contrast; right: T2-weighted). The user switches the sidebar to Navigation mode and schedules a capture batch directly from the series list: orthogonal MPRs (axial/coronal/sagittal) for the T1 post-contrast series and a native 2D axial slice batch (non-MPR) for the T2 series. After confirming enqueue, the system generates model-ready images via offscreen re-rendering from serialized viewing state and returns to Chat mode, showing progressive thumbnail accumulation as captures complete. The user then submits the prompt ("describe the findings concisely") together with the staged images; after upload and remote inference, the model's answer is streamed into the chat transcript. Imaging is sourced from the UPenn-GBM dataset (TCIA).
